# Attention to Progression Principles and Variables of Exercise Prescription in Workplace-Related Resistance Training Interventions: A Systematic Review of Controlled Trials

**DOI:** 10.3389/fpubh.2022.832523

**Published:** 2022-03-25

**Authors:** Gerrit Stassen, Lukas Baulig, Ole Müller, Andrea Schaller

**Affiliations:** Working Group Physical Activity-Related Prevention Research, Institute of Movement Therapy and Movement-Oriented Prevention and Rehabilitation, German Sport University Cologne, Cologne, Germany

**Keywords:** health promotion, workplace, resistance training, reporting, transfer

## Abstract

**Background:**

The workplace is an important setting for adult health promotion including exercise training such as resistance training (RT). Since the reporting of exercise training interventions is generally inconsistent, the objective of this systematic review was to investigate the attention to principles of RT progression and variables of RT exercise prescription in workplace-related RT interventions.

**Methods:**

A systematic literature search was conducted in the databases LIVIVO, PubMed, SPORTDiscus, and Web of Science (2000–2020). Controlled trials with apparently healthy “employees” and a main focus on RT were included. RT principles and variables were extracted and rated by two reviewers (reported, not reported, or unclear). Sum scores for each RT intervention and percentages regarding each principle and variable were calculated.

**Results:**

Overall, 21 articles were included (18 primary studies, 3 protocols). Summarized narratively, the interventions showed different positive effects on strength- or performance-related and/or health- or complaint-related outcomes. The reporting of the RT principles and variables was varied [progressive overload: 94% of the studies, specificity: 78%, variation (periodization): 39%, muscle action: 94%, loading: 94%, volume; 67%, exercise selection: 89%, exercise order: 47%, rest periods between sets: 33%, rest periods between exercises: 27%, repetition velocity: 44%, and frequency: 100%].

**Conclusion:**

Several key RT principles and variables were reported inconsistently, reducing reproducibility and pointing to the need for standardized RT intervention reporting in workplace-related interventions. Exercise science and workplace promotion should be further linked, since accurate reporting is a prerequisite for transferring robust findings into practice.

## Introduction

For years, the workplace has developed into an important setting regarding adult health promotion, as it is one setting of everyday life where health is created and lived (1). Since working adults spend almost half of their waking hours at work (2), large and diverse groups of the population can be reached there. Accordingly, the workplace also offers an environment for health-promoting exercise training (3).

In general, the multiple positive health benefits of physical activity (PA) and fitness training are well documented (4–8). In this respect, workplace-related PA interventions conducted directly at the workplace and, where appropriate, also during working hours, have shown positive effects on, e.g., activity behavior, physical fitness and cardio-metabolic health, musculoskeletal disorders, and the prevention of work-related pain (9–13). They improve overall well-being and work ability, reduce sickness absenteeism and sick leave, and can generate a positive financial return (14–16).

Besides the general health-promoting effects of PA, scientific findings emphasize the independent positive effects of resistance training (RT) on health (17, 18). RT attenuates the age-related decrease in muscle mass and strength (19, 20), improves health-related quality of life (21), and there is an inverse association of muscular strength and fitness with all-cause mortality, even after adjusting for cardiorespiratory fitness or proven risk factors (22–25). Considering the workplace setting, the “medicine” RT (26, 27) shows inter alia positive effects on physical (e.g., pain reduction) and work-related (e.g., productivity) factors of employees (28–33).

However, the mode of exercise training seems to be decisive for achieving positive effects, which is why optimal planning is essential (33). The proper application of training principles leads to improvements of components of fitness or health through physical adaptions. In order to optimize effectiveness so that improvements occur, targeted (resistance) training is supposed to be designed according to basic principles (34–36). A RT program should be systematically altered according to the foremost principles of RT progression to make the body adapt to changing stimuli: progressive overload, specificity, and variation (periodization) (37, 38). Furthermore, proper RT exercise prescription involves several key variables: muscle action, loading, volume, exercise selection, exercise order, rest periods between sets and exercises, repetition velocity, and frequency (37–39). Nevertheless, systematic reviews show that the application and reporting of principles and guidelines of exercise training is inconsistent and could be improved in intervention studies in general (40–45) and in RT studies in particular (46–48).

Given the great potential of workplace-related interventions to reach diverse adult target groups in the context of health promotion, RT interventions in this setting should be reported as comprehensively as possible to facilitate replicability and thus transfer of promising approaches. Thus, based on the outlined state of research, the question of this systematic review was: How are the principles of RT progression and variables of RT exercise prescription applied in studies involving workplace-related RT interventions?

## Methods

This systematic review was conducted in accordance with the PRISMA-recommendations (49, 50).

### Inclusion/Exclusion Criteria

The studies were selected according to the PICOS criteria (participants, interventions, comparators, outcomes, study design) (50) as well as setting, language and time frame.

Intervention studies with apparently healthy “employees” (defined as working-age adults in full- or part-time-employment) without restrictions regarding sex or job/type of occupation were taken into account, while studies with specific patient populations or populations focusing on specific diseases or comorbidities were not. To be included, studies must have examined an intervention with at least one study arm with main focus on RT (mentioned in the rationale, hypothesis and/or methods section) conducted within the workplace-context and/or at the workplace (“workplace-related”) and had other training forms included for warm-up or cool-down only. Studies with alternative PA interventions without a main RT focus (general fitness training, other training forms, mixed PA interventions) and multicomponent interventions were excluded. Due to the review focus, no restrictions were placed on the comparison groups (no intervention, non-RT intervention, minimal intervention, waitlist control etc.) and the outcome measurements.

Therefore, any prospective research study (experimental design) with a workplace-related RT intervention and a comparator group [randomized controlled trials (RCTs), Cluster RCTs, controlled trials] with no limitation on the length of the intervention or a follow-up was considered. RCTs are generally the most powerful experimental design but to include them alone may be too restrictive to investigate workplace-related RT interventions as many studies occur in naturalistic workplace settings where RCTs are not always possible (51). The language limitation was English or German and the time limitation was from January 2000 to December 2020 due to past developments in the field of health promotion and especially workplace health promotion [e.g., Jakarta Declaration on Leading Health Promotion into the 21st Century (52) and Luxembourg Declaration on Workplace Health Promotion (53)].

### Information Sources and Search

A computerized systematic literature search was conducted in LIVIVO, PubMed, SPORTDiscus, and Web of Science at the end of January 2021. Search terms related to workplace interventions (e.g., “worker^*^,” “employe^*^,” and “workplace”), RT (e.g., “resistance,” “weight,” or “strength” and “training” or “exercise”) and controlled trials (e.g., “controlled study” OR “RCT”) were used with operators (“OR,” “AND,” and “NOT) and truncations (“^*^”) with appropriate adjustments for each database (Supplementary Table 1).

### Study Selection

Articles were imported into the literature management program Rayyan (54). After removing all duplicates, two reviewers (GS and LB) independently screened all titles/abstracts in a first step and full texts in a second step based on the inclusion/exclusion criteria. Any disagreements were resolved by consensus or consulting with a third reviewer (OM). If the full texts were secondary analyses, the corresponding primary studies were additionally searched for and included instead (if eligible and not already included). If underlying study protocols were mentioned, these were additionally used for data collection regarding the RT intervention.

### Data Collection and Items

On a first constructed form, the following study characteristics were extracted: name of first author and year, study design, study sample [including occupation description, baseline sample size, sex, and age (years)], RT intervention (general description of all study interventions and period, frequency and duration, location, and supervision of the RT study arm). In addition, significant effects of the included RT interventions were listed with respect to the control group with a focus on the intervention period only (without considering possible follow-ups).

Using a second constructed form, principles of RT progression (37, 38) and variables of RT exercise prescription (37–39) were extracted from the methods sections of the included articles or additionally from the underlying study protocols, respectively, and rated independently by two reviewers (GS and LB) according to the working understandings (Tables 1, 2). Principles and variables were rated as follows: yes (+) “reported/applied,” no (−) “not reported/not applied,” or unclear (?) if it was unclear or inconsistent whether a principle/variable was reported/applied. “Not applicable” (na) was recorded for “exercise order” and “rest between exercises” if only one exercise was used and for “rest between sets” if only single sets were used. Disagreements were resolved through personal communication or consulting with a third reviewer (OM).

**Table 1:** Principles of resistance training progression (working understandings).

**Principle**	**Working understanding**
Progressive overload (prog over)	Gradually and systematically increasing the stress on the body during training by changing one or more training variables, which is necessary for further improvement
Specificity (spec)	Specificity is the physiological adaptation to the type of stimulus applied, which is why effective programmes are designed to target-specific training goals
Variation (periodization) (per)	Training variation, or periodization, describes the systematic process of making changes to one or more program variables over time to keep the training stimulus challenging and effective
Classical	High training volume and low intensity at the beginning, while in the course of the training the volume decreases and the intensity increases
Reverse	Intensity is initially at its highest and volume at its lowest, while the intensity decreases and the volume increases as the training progresses
Undulating	Allows variation of volume and intensity by rotating different protocols to train different components of neuromuscular performance within one cycle

*Compare Ratamess et al. (37) and Kraemer and Ratamess (38)*.

**Table 2:** Variables of resistance training exercise prescription (working understandings).

**Variable**		**Working understanding**
Muscle action (m act)		Most resistance training programs primarily involve dynamic repetitions with both concentric and eccentric muscle actions (whereas isometric actions play a secondary role)
Loading (load)		Proper loading increase follows e.g. one or more of the following schemes: based on a percentage of the one-repetition maximum, based on a targeted repetition number, or within a prescribed repetition zone
Volume (vol)		Summation of the total number of repetitions and sets performed during one training session (thus also determined by the number of exercises)
Exercise selection (ex sel)		Selection based on multiple modalities (single- and multiple-joint, unilateral and bilateral, and, e.g., free weights or machines etc.) with corresponding exercise specifications
Exercise order (ex ord)		Sequence of exercises, e.g., using a precise scheme (whole-body training, upper/lower body split training and muscle group split training)
Rest periods (r per)	Between sets (bet s)	Varying according to the complexity of the exercise, the training goal (objective for incorporating the exercise into the program) and the training status of the individual
	Between exercises (bet ex)	
Repetition velocity (vel)		Speed at which dynamic exercises are performed, which is given in seconds and the relationship is between the concentric and eccentric phases
Frequency (freq)		Frequency describes the number of workouts within a period of time (depending on several factors such as intensity, volume, training level, training goals, and recovery ability)

*Compare Ratamess et al. (37), Kraemer and Ratamess (38), and Bird et al. (39)*.

### Study Quality

All included studies were subjected to the Effective Public Health Practice Project's (EPHPP) quality assessment tool for quantitative studies (55, 56). Two reviewers (LB and OM) independently assessed the quality of the studies reaching consensus through discussion [consulting a third reviewer in case of uncertainties (GS)]. According to the EPHPP dictionary (57), the first six components were included in the assessment (selection bias, study design, confounders, blinding, data collection methods, and withdrawals and dropouts) and rated as weak, moderate or strong. Since it is impossible to blind participants and instructors in RT studies, blinding according to the EPHPP is assessed on two levels: whether the outcome assessor(s) were aware of the intervention or exposure status of participants and whether the study participants were aware of the research question (55). The assessment is based on the extent to which both, one or none are fulfilled (57).

### Data Analysis and Synthesis of Results

The significant effects of the study arm(s) with RT in the intervention studies (pre-post, compared to the control group) were summarized narratively.

Reported/applied (+) principles of RT progression and variables of RT exercise prescription were given a score of “1,” not reported/not applied (−) and unclear (?) a “0,” and not applicable (na) no score. Sum scores of progression principles and exercise prescription variables were calculated and corrected for the number of “na.” Percentages of RT intervention descriptions reporting/applying each principle and variable were also calculated (proportion relative to the total number).

## Results

### Study Selection

**Figure 1:**
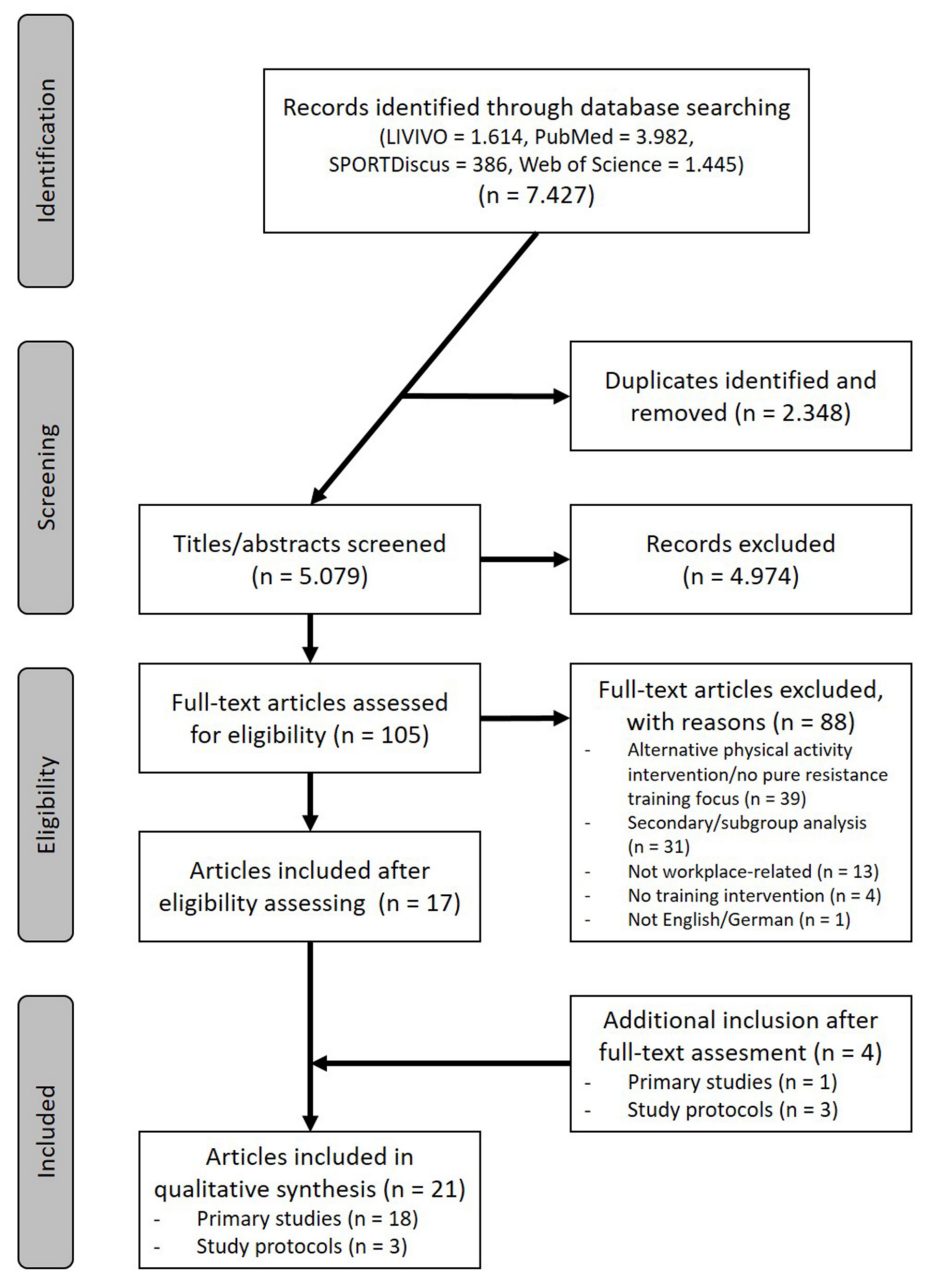
Flow diagram illustrating the search and selection process.

The systematic search resulted in 7.427 articles and after removing duplicates and screening titles/abstracts a total of 105 potentially relevant articles were assessed for eligibility. Based on the full-text assessments, 1 additional primary study and 3 study protocols were added, resulting in 21 articles remaining for the qualitative synthesis (18 studies, 3 protocols) (Figure 1).

### Study Characteristics

Table 3 presents the characteristics of the 18 included primary studies. The majority were RCTs [9 studies (58, 59, 64–67, 69, 70, 73, 74)] and another described as feasibility study (71). Furthermore, there was one non-randomized CT (62) and 6 cluster RCTs (60, 61, 63, 68, 72, 75), with 2 each evaluating a subgroup of the intervention groups in an overarching study (60, 63).

**Table 3:** Included primary studies.

**No**	**References**	**Study design**	**Study sample**		**Intervention**		**Significant effects (RT, pre-post-intervention)**	**Study quality** **(EPHPP)^a^**
			**Occupation**	**Total: a) baseline sample size, b) sex, c) age (years)** **Intervention groups: a) baseline sample size, b) sex, c) age (years)**	**General descriptions of interventions**	**RT study arm(s):** **a) intervention period** **b) frequency and duration** **c) location** **d) supervision**		
1	Andersen et al. (58)	Randomized controlled trial	Employees with monotonous and repetitive tasks (computer work common) and chronic neck muscle pain	Total: a) *n* = 48, b) 100% ♀, c) not sp. IG1:a) *n* = 18, b) 100% ♀, c) 44 ± 9 IG2: a) *n* = 16, b) 100% ♀, c) 45 ± 9 CG: a) *n* = 14, b) 100% ♀, c) 42 ± 8	IG1: high-intensity specific strength training for the neck and shoulder muscles (dumbbell exercises) IG2: high-intensity general fitness training (bicycle ergometer) CG: health counseling on a group level and an individual level, not offered any physical training	a) 10 weeks b) 3 × 20 min/week c) at the workplace d) supervised	Muscle strength (shoulder) ↑ Trapezius muscle pain (general pain since the last training session) ↓ Trapezius muscle pain (worst pain since the last training session) ↓	Weak
2	Andersen et al. (59)	Randomized controlled trial	Employees (white-collar organizations) with frequent neck/shoulder pain	Total: a) *n* = 198, b) 88% ♀, c) not sp. IG1: a) *n* = 66, b) 88% ♀, c) 44 ± 11 IG2: a) *n*= 66, b) 88% ♀, c) 42 ± 11 CG: a) *n* = 66, b) 88% ♀, c) 43 ± 10	IG1: resistance training (shoulder abductions/lateral raises with elastic bands) (2 min sessions) IG2: resistance training (shoulder abductions/lateral raises with elastic bands) (12 min sessions) CG: information on general health (weekly e-mailed information and provision of internet links)	a) 10 weeks b) 5 × 2 or 5 × 12 min/week, respectively c) at the workplace d) unsupervised (initial training instruction and explanation, optional help available)	Muscle strength (shoulder) ↑ (both IGs) Neck/shoulder pain intensity ↓ (both IGs) Neck/shoulder muscle tenderness ↓ (both IGs)	Moderate
3	Andersen et al. (60)	Cluster randomized controlled trial	Office workers (national public administrative authority) with and without neck and/or shoulder pain	Total: a) *n* = 449, b) 62% ♀, c) 46 ± 10 IG1: a) *n* = 116, b) 62% ♀, c) 47 ± 10 IG2: a) *n*= 126, b) 69% ♀, c) 46 ± 10 IG3: a) *n*= 106, b) 58% ♀, c) 45 ± 10 CG: a) *n* = 101, b) 58% ♀, c) 46 ± 10	IG1: specific strength training for the neck and shoulder muscles (dumbbell exercises) (once/week) IG2: same as IG1 (3 times/week) IG2: same as IG1 (9 times/week) CG: not offered any physical training	a) 20 weeks b) 1 × 60 or 3 × 20 or 9 × 7 min/week, respectively. c) at the workplace d) every other training session supervised	Neck and shoulder pain intensity (last 3 months) ↓ (all three IGs) Work disability ↓ (IG1 and IG2)	Weak
4	Blangsted et al. (61)	Cluster randomized controlled trial	Office workers (public administration)	Total: a) *n* = 549, b) 64% ♀, c) n.r. IG1: a) *n* = 180, b) 70% ♀, c) n.r. IG2: a) *n* = 187, b) 64% ♀, c) n.r. CG: a) *n* = 182, b) 59% ♀, c) n.r.	IG1: specific resistance training for the muscles in the shoulder and neck region (dumbbells, static exercises, rowing and kayaking ergometer) IG2: all-round physical exercise (physical exercises introduced at the worksite, mixture of activities) CG: encouraged to form groups to improve existing nonoptimal health and work conditions (not performing additional physical activity)	a) 12 months b) 3 × 20 min/week c) at the workplace d) 2/3 supervised	Neck and shoulder symptoms intensity (last 3 months) ↓ Neck and shoulder symptoms duration (last 3 months) ↓	Weak
5	Escriche-Escuder et al. (62)	Non-randomized controlled trial	Hospital porters (university clinic) with at least one episode of musculoskeletal pain during the last month	Total: a) *n* = 37, b) 73% ♀, c) n.r. IG: a) *n* = 19, b) 89% ♀, c) 53 ± 9 CG: a) *n* = 18, b) 56% ♀, c) 49 ± 11	IG: brief whole-body resistance training in groups in circuit (elastic band and body weight exercises) CG: maintenance of usual activity	a) 9 weeks b) 5 × 15 min/week c) site close to the workplace d) supervised	Muscle strength (push-ups) ↑ Back muscular endurance ↑ Pain overall status ↓ Pain intensity (hips/thighs and ankles/feet, last three months) ↓ Well-being ↑ Work satisfaction ↑ Work impairment ↓ Desire exercising ↑ Energy ↑	Moderate
6	Gram et al. (63)	Cluster randomized controlled trial	Office workers (national public administrative authority) [same target group as in (60)]	Total: a) *n* = 351, b) 62% ♀, c) n.r. IG1: a) *n* = 126, b) 69% ♀, c) 46 ± 10 IG2: a) *n* = 124, b) 58% ♀, c) 45 ± 11 CG: a) *n* = 101, b) 58% ♀, c) 46 ± 10	IG1: specific strength training for the neck and shoulder muscles (dumbbell exercises), regularly supervised [same group as IG2 in (60)] IG2: same as IG1, minimally supervised CG: not offered any physical training	a) 20 weeks b) 3 × 20 min/week c) at the workplace d) every other training session supervised or minimally supervised (only initial instructions for two sessions), respectively	Neck pain intensity (last 7 days) ↓ (IG2 vs. CG) Headache intensity (last month) ↓ (both IGs) Days with headache (last month) ↓ (both IGs)	Weak
7	Haufe et al. (64)	Randomized controlled trial	Employees from medium-sized companies (desk work and manufacturing)	Total: a) *n* = 226, b) 60% ♂, c) 43 ± 10 IG: a) *n* = 112, b) 57% ♂, c) 44 ± 10 CG: a) *n* = 114, b) 62% ♂, c) 42 ± 11	IG: exercises without equipment particularly for the trunk musculature CG: asked to continue the current lifestyle	a) 5 months b) 3 × 20 min/week c) at the workplace or at home d) initial instruction and supervision once a month	Muscle strength (back extension) ↑ Low back pain (last 7 days) ↓	Moderate
8	Helmhout et al. (65)	Randomized controlled trial	Army employees with non-specific low back pain (longer than 12 weeks)	Total: a) *n* = 81, b) 100% ♂, c) n.r. IG: a) *n* = 41, b) 100% ♂, c) 41 ± 10 CG: a) *n* = 40, b) 100% ♂, c) 40 ± 9	IG1: high-intensity training program of the isolated lumbar extensor muscle groups (lower back machine) CG: non- progressive, low-intensity resistance protocol (lower back machine) (below strength training stimulus)	a) 12 weeks b) 1 × 5-10 min/week (weeks 1-2) and 2 × 5-10 min/week (weeks 3-12), respectively c) associated training/therapy department d) supervised	Comparable positive effects in both IG and CG (functional disability due to low back pain, self-experienced health, fear of movement) with back extension strength ↑ in IG	Moderate
9	Helmhout et al. (66)	Randomized controlled trial	Army employees (predominantly male soldiers) with non-specific non-acute low back pain	Total: a) *n* = 127, b) 97% ♂, c) not sp. IG: a) *n* = 71, b) 97% ♂, c) 37 ± 11 CG: a) *n* = 56, b) 96% ♂, c) 35 ± 11	IG: lumbar extensor strength training program (lower back machine) CG: regular physical therapy (exercise therapy and aerobic activities)	a) 10 weeks b) 2 × 5-10 min/week c) associated training/therapy department d) supervised	Positive effects comparable to those of CG (on back extension strength, low-back specific functional status, patient-specific functional status, and global perceived effect)	Moderate
10	Li et al. (67)	Randomized controlled trial	Employees (monotonous jobs, daily computer use) with chronic work-related neck pain	Total: a) *n* = 109, b) 100% ♀, c) n.r. IG1: a) *n* = 38, b) 100% ♀, c) 36 ± 8 IG2: a) *n* = 35, b) 100% ♀, c) 34 ± 9 CG: a) *n* = 36, b) 100% ♀, c) 34 ± 8	IG1: neck resistance training (progressive) (elastic bands) IG2: neck resistance training (fixed load) (elastic bands) CG: health-related information, discussions, and presentations	a) 6 weeks b) at least 3 times/week (duration n.r.) c) both at the workplace and at home d) supervision once a week	Neck muscle strength (flexion, extension, lateral flexion) ↑ (both IGs) Pain intensity ↓ (both IGs, greater effect in IG1) Pain threshold ↓ (both IGs) Neck disability ↓ (both IGs)	Strong
11	Mayer et al. (68)	Cluster randomized controlled trial	Full-duty career firefighters (fire stations of a municipal fire department)	Total: a) *n* = 96, b) 91% ♂, c) 35 ± 9 IG: a) *n* = 54, b) 82% ♂, c) 38 ± 10 CG: a) *n* = 42, b) 96% ♂, c) 31 ± 8	IG: exercise (mat-based core exercises and back extension exercise on a Roman chair) plus usual physical fitness routine CG: usual physical fitness routine alone	a) 24 weeks b) 2 × 10 min/week c) at the workplace d) supervised	Back muscular endurance ↑ Core muscular endurance ↑	Moderate
12	Mulla et al. (69)	Randomized controlled trial	Office employees (automotive industry)	Total: a) *n* = 43, b) 63% ♀, c) n.r. IG: a) *n* = 21, b) 57% ♀, c) 44 ± 11 CG: a) *n* = 22, b) 68% ♀, c) 43 ± 10	IG: leg- strengthening classes (exercises to target major muscle groups) CG: maintenance of usual activity	a) 12 weeks b) 3 × 45 min/week c) on site gymnasium/fitness center d) supervised	Lower extremity functionality ↑ Mobility ↑ (walk test ↓, stair climbing test ↓)	Moderate
13	Muñoz-Poblete et al. (70)	Randomized controlled trial	Manufacturing workers exposed to excessive effort and repetitive tasks principally with the upper limbs (furniture manufacturing)	Total: a) *n* = 109, b) not sp., c) not sp. IG: a) *n* = 52, b) 79% ♂, c) 29 ± 5 CG: a) *n* = 53, b) 83% ♂, c) 28 ± 5 (data only available for completers)	IG: resistance-based exercise program for the upper limbs (elastic bands) CG: stretching exercises	a) 16 weeks b) 3 × 15 min/week c) at the workplace d) supervised	Upper limb pain intensity ↓ Work functionality ↑	Moderate
14	Nygaard Andersen et al. (71)	Randomized controlled feasibility study	Professional symphony orchestra musicians	Total: a) *n* = 23, b) 61% ♀, c) n.r. IG: a) *n* = 12, b) 67% ♀, c) 45 ± 11 CG: a) *n* = 11, b) 55% ♀, c) 47 ± 8	IG: high-intensity specific strength training, focusing on the neck and shoulder muscles (dumbbell exercises) CG: high-intensity general fitness training for the legs only (bicycle ergometer)	a) 9 weeks b) 3 × 20 min/week c) at the workplace or at home d) supervised	Pain intensity (last 7 days) ↓	Weak
15	Sjögren et al. (72)	Cluster randomized controlled trial (cross-over)	Office workers (public administration)	Total: a) *n* = 90, b) 73% ♀, c) 46 ± 9 IG: a) *n* = 55, b) 84% ♀, c) n.r. CG: a) *n* = 35, b) 57% ♀, c) n.r.	IG1: light resistance training (six dynamic symmetrical movements, air resistance equipment) CG: same as IG after 15 week no-intervention (cross over)	a) 15 weeks b) 5 × 6 min/week (weeks 1-5) and 7-8 × 8 min/week (weeks 6-15), respectively c) training facility of the workplace d) non-supervised (guidance in three group sessions at 5-week intervals)	Subjective physical well-being ↑	Strong
16	Sundstrup et al. (73)	Randomized controlled trial	Slaughterhouse workers with chronic pain in the shoulder, elbow/forearm, or hand/wrist, and work disability	Total: a) *n* = 66, b) 77% ♂, c) n.r. IG: a) *n* = 33, b) 76% ♂, c) 48 ± 9 CG: a) *n* = 33, b) 79% ♂, c) 43 ± 9	IG: high-intensity resistance training for the shoulder, arm, and hand muscles (small training equipment) CG: ergonomic training and education	a) 10 weeks, b) 3 × 10 min/week c) at the workplace d) supervised	Muscle strength (wrist and shoulder) ↑ Pain intensity (shoulder, elbow/forearm, and hand/wrist) (last 7 days) ↓ Work disability ↓	Strong
17	Zavanela et al. (74)	Randomized controlled trial	Bus drivers	Total: a) *n* = 132, b) 100% ♂, c) n.r. IG: a) *n* = 60, b) 100% ♂, c) n.r. CG: a) *n* = 72, b) 100% ♂, c) n.r.	IG: resistance training (whole-body program) CG: maintaining normal daily activities	a) 24 weeks b) 3 times/week (weeks 1-8) and 4 times/week (weeks 9-24) (duration n.r.), respectively c) on site gymnasium/fitness center d) supervised	Muscle strength (bench press, leg press) ↑ Muscular endurance (sit-ups, push-ups, trunk flexibility) ↑ Pain incidence (back, legs, arms, shoulders, and head) (last 2 weeks) ↓ Blood pressure ↓ Worker abseentism ↓	Weak
18	Zebis et al. (75)	Cluster randomized controlled trial	Industrial workers (laboratory technicians, repetitive tasks and data processing)	Total: a) *n* = 537, b) n.r., c) n.r. IG: a) *n* = 282, b) 89% ♀, c) 42 ± 11 CG: a) *n* = 255, b) 80% ♀, c) 42 ± 10	IG: high-intensity strength training for the neck and shoulders (dumbbell exercises) CG: advice to stay physically active and supervisor consultation (once a week)	a) 20 weeks b) 3 × 20 min/week c) at the workplace d) every other training session supervised	Neck pain intensity (last 7 days) ↓	Moderate

*IG, intervention group; CG, control group; n.r., not reported.↑ or ↓, outcome increased or decreased (p ≤ 0.05)*.

a *See Supplementary Table 2 for the quality assessment of all EPHPP components*.

The target groups were diverse in terms of types of occupation, with employees in inactive work environments (e.g., white collar/office desk work, laboratory technicians), physically active jobs (hospital porters, firefighters, army employees, musicians) or also blue collar workers (manufacturing, slaughterhouse, bus drivers). Some studies specified eligible participants in terms of health status or physical complaints (e.g., neck/shoulder or low back pain) whereas others targeted employees generally. The baseline sample size in the studies ranged from 23 (71) to and 549 (61) participants, with four studies including females only (58, 67) or predominantly females (>80%) (59, 75) and 5 studies males only (65, 74) or predominantly males (66, 68, 70), respectively. The majority of studies had, on average, middle-aged samples.

The RT interventions used different materials or equipment, such as dumbbells, elastic bands or machines, in different organizational forms [ranging from, e.g., training consisting of only one exercise or even one single set (59, 65, 66) to circuit training in groups (62)] with the sessions being supervised to varying extents (fully, alternating to minimal, and not at all). The majority of studies were conducted directly at the workplace, although three studies had the option of exercising at home (64, 67, 71). Interventions that included training in groups and/or used larger equipment took place in associated training/therapy departments (65, 66) or in fitness centers/training facilities within the workplace (69, 72, 74) or in close proximity (62), respectively. The intervention periods were between 6 weeks (67) and 12 months (61) with a frequency of at least 1-2 times/week (60, 65) up to sometimes several times/day (60, 72), with most interventions conducting RT 2-3 times/week.

### Study Quality and Results

Overall, with 9 out of 18 studies, the majority of studies were rated as moderate (59, 62, 64–66, 68–70, 75), six studies were again rated as weak (58, 60, 61, 63, 71, 74), and the remaining three studies were rated as strong (67, 72, 73) (Table 3). In summary, study design and data collection methods were rated as the strongest across all studies, while the distribution of the other components was more varied (weak/moderate/strong) (Supplementary Table 2).

Within the workplace-related RT interventions, different positive effects (pre-post-intervention) were reported (Table 3).

Significant improvements in (muscular) strength- or performance-related outcomes as a result of RT intervention were reported in 10 studies. Upper limb strength (neck, shoulder, or wrist) after an specific RT program was examined in four studies (58, 59, 67, 73). Comparable to this, four studies examined the strength/endurance of the back or trunk (64–66, 68). The other two studies performed multi-joint exercises to assess strength/performance effects to correspond to the whole-body training conducted in the interventions (62, 74).

Significant improvements regarding pain- or complaint-related outcomes were reported in 13 studies. Most frequently, the intensity and/or duration of neck or upper back/limb pain or headache was examined (eight studies) (58–61, 63, 70, 73, 75), often using variants of a visual analog scale. The other studies assessed either complaints in the lower back or lower extremities or a general pain condition without specifying more precisely the body region (62, 64, 67, 71, 74).

Further different outcomes assessed for which significant improvements were shown included muscle tenderness (59), functional status or mobility (65, 66, 69), well-being (62, 72), work-related outcomes such as disability, functionality, satisfaction, impairment, or abseentism (60, 62, 70, 73, 74).

In two studies, pre-post improvements also occurred in the control groups, as these were a comparison with regular physiotherapy (66) or a comparison with low-intensity strength training, respectively, although in the latter study, only the high-intensity intervention group also showed significant effects regarding strength (65).

### Resistance Training Intervention Reporting

The rating of the RT interventions in terms of attention to principles of progression and variables of exercise prescription is listed in Table 4 and reported in detail in Supplementary Table 3. For the assessment, protocols could also be taken into account for 4 of the 18 primary studies, as one protocol (76) refers to the two aforementioned studies that each evaluate a subset of the intervention groups in an overarching study (60, 63).

**Table 4:** Application of principles of progression and variables of exercise prescription.

**No**	**References**	**Principle**			**Sum score^a^**	**Variable**									**Sum score^b^**
		**Prog over**	**Spec**	**Per**		**M act**	**Load**	**Vol**	**Ex sel**	**Ex ord**	**R per**		**Vel**	**Freq**	
											**Bet s**	**Bet ex**			
1	Andersen et al. (58)	(+)	(+)	(+)	3/3	(+)	(+)	(+)	(+)	(+)	(–)	(–)	(?)	(+)	6/9
2	Andersen et al. (59)	(+)	(+)	(–)	2/3	(+)	(+)	(+)	(+)	(na)	(+)	(na)	(+)	(+)	7/7
3	Andersen et al. (60) [protocol: (76)]	(+)	(+)	(+)	3/3	(+)	(+)	(?)	(+)	(+)	(+)	(–)	(–)	(+)	6/9
4	Blangsted et al. (61)	(+)	(+)	(–)	2/3	(+)	(+)	(+)	(+)	(?)	(–)	(–)	(–)	(+)	5/9
5	Escriche-Escuder et al. (62)	(+)	(?)	(+)	2/3	(+)	(+)	(+)	(+)	(+)	(na)	(+)	(+)	(+)	8/8
6	Gram et al. (63) [protocol: (76), same as (60)]	(+)	(+)	(+)	3/3	(+)	(+)	(?)	(+)	(+)	(+)	(–)	(–)	(+)	6/9
7	Haufe et al. (64)	(–)	(+)	(–)	1/3	(+)	(?)	(?)	(?)	(–)	(–)	(–)	(?)	(+)	2/9
8	Helmhout et al. (65)	(+)	(+)	(?)	2/3	(+)	(+)	(+)	(+)	(na)	(na)	(na)	(+)	(+)	6/6
9	Helmhout et al. (66) [protocol: (77)]	(+)	(+)	(–)	2/3	(+)	(+)	(+)	(+)	(na)	(na)	(na)	(+)	(+)	6/6
10	Li et al. (67)	(+)	(+)	(–)	2/3	(+)	(+)	(+)	(+)	(?)	(na)	(–)	(+)	(+)	6/8
11	Mayer et al. (68)	(+)	(+)	(–)	2/3	(+)	(+)	(+)	(+)	(?)	(na)	(+)	(+)	(+)	7/8
12	Mulla et al. (69)	(+)	(?)	(?)	1/3	(+)	(+)	(–)	(?)	(–)	(–)	(–)	(–)	(+)	3/9
13	Muñoz-Poblete et al. (70)	(+)	(+)	(–)	2/3	(+)	(+)	(–)	(+)	(?)	(–)	(?)	(+)	(+)	5/9
14	Nygaard Andersen et al. (71)	(+)	(+)	(+)	3/3	(+)	(+)	(+)	(+)	(+)	(–)	(–)	(–)	(+)	6/9
15	Sjögren et al. (72)	(+)	(?)	(–)	1/3	(+)	(+)	(+)	(+)	(+)	(na)	(+)	(+)	(+)	8/8
16	Sundstrup et al. (73) [protocol (78)]	(+)	(+)	(+)	3/3	(+)	(+)	(+)	(+)	(+)	(–)	(–)	(?)	(+)	6/9
17	Zavanela et al. (74)	(+)	(?)	(–)	1/3	(?)	(+)	(+)	(+)	(?)	(+)	(+)	(–)	(+)	6/9
18	Zebis et al. (75)	(+)	(+)	(+)	3/3	(+)	(+)	(?)	(+)	(?)	(–)	(–)	(?)	(+)	4/9
Proportion		94% (17/18)	78% (14/18)	39% (7/18)	70% (38/54)	94% (17/18)	94% (17/18)	67% (12/18)	89% (16/18)	47% (7/15)	33% (4/12)	27% (4/15)	44% (8/18)	100% (18/18)	69% (103/150)

*prog over, progressive overload; spec, specificity; per, variation (periodization); m act, muscle action; load, loading; vol, volume; ex sel, exercise selection; ex ord, exercise order; r per, rest periods (bet s = between sets, bet ex, between exercises); vel, repetition velocity; freq, frequency; RT, resistance training. See Supplementary Table 3 for the citations or text passages on which the decisions were based. Note:*

a *Possible maximum score = 3*.

b *Possible maximum score = 9 (depending on the correction for the number of “na”)*.

#### Principles of Resistance Training Progression

Overall, 70 % of the principles of RT progression (Table 1) were reported in the included studies (38/54), ranging from 1 to 3 principles with 6 of the 18 studies reporting all 3 principles (58, 60, 63, 71, 73, 75) (Table 4). The principle of progressive overload was explicitly stated in all but one study (94%, 17/18). Specificity, in turn, was documented for 78% (14/18), whereby the four studies classified as “unspecific” were rated as “unclear” as they reasoned whole-body trainings with its impact on, among other things, musculoskeletal pain (62), subjective physical well-being (72) or absenteeism (74), or investigated a specific osteoarthritis training in a general target group (69). The least documented principle is variation understood as systematic variation of both intensity and volume over the course of training (periodization) (Table 1) with 39% (7/18) describing classical (58, 62, 71, 73) or classical/undulating (60, 63, 75) models, respectively. Two studies were rated as unclear because in the first case, training was only performed at a lower intensity and a higher volume in the first 2 of the 12 intervention weeks and the change after week 3 does not appear to be systematic (65) [moreover, the same research group did not apply any periodization in another included subsequent study (66)] and in the second case, although an increase in volume is indicated to some extent by increased duration and number of repetitions, no change in intensity is described (69).

#### Variables of Resistance Training Exercise Prescription

Overall, 69% of all variables of resistance training exercise prescription (Table 2) were reported in the included studies (103/150, corrected for the number of “not applicable”) (Table 4). The variable muscle action is reported in 94% (17/18), describing concentric, eccentric as well as isometric muscle action, with the only missing studies most likely using dynamic exercises but not explicitly stating this (74). Loading is applied in 94% (17/18) and was predefined as, e.g., prescribed repetition range for an exercise or a session (59, 65, 66, 68), as a percentage of a certain repetition maximum or the maximal strength (58, 60–63, 67, 71, 73–75), based on a rating of perceived exertion (69, 72), or as a fixed weight during a defined intervention phase (70). The only “unclear” study indicated load adjustments by therapists on site without explaining the procedure in more detail (64). The training volume, which can be recognized by an indication of the number of exercises, sets and repetitions, was given in 67% (12/18). The exercise selection was named in 89% (16/18) of the studies, while the exact exercise order was only clearly described in 47% (7/15) (corrected for single exercise interventions), as in some cases it remained unclear whether the order or numbering in the article texts remained the same throughout the intervention phase. Corrected for single exercise interventions and single set interventions, rest periods between exercises and between sets were also reported comparatively infrequently at 33% (4/12) and 27% (4/15) respectively, which also applies to the repetition velocity (given in seconds) [44% (8/18)]. Finally, all studies 100 % (18/18) applied the frequency of the training sessions (compare also Table 3).

## Discussion

To the best of our knowledge, this review is the first to specifically address attention to progression principles and variables of exercise prescription in workplace-related RT interventions. The main finding of the analysis was that several important RT principles and variables were inconsistently reported. While the goal of this systematic review was not to evaluate whether general RT recommendations were followed, the results do highlight some gaps in reporting (and potential for intervention planning).

The principle of progressive overload was mentioned in almost all studies indicating that there is agreement on the importance of increasing the stress to achieve adaptations and improvements. Due to the often limited time available for interventions at the workplace (79, 80), progressive overload provides the foremost basis for effective but also efficient interventions.

The principle of specificity was rated as unclear in four studies, as these (62, 69, 72, 74) used a more general training approach. Specific training should ensure that the most optimal type of exercise is chosen for a desired outcome (e.g., specific neck and shoulder training to prevent or reduce pain). Nonetheless, non-specific whole-body training could be reasoned from a primary prevention perspective since regular RT has a wide range of health-enhancing effects (17, 26, 81, 82). Depending on possibly rather broader intervention goals in the context of workplace health promotion (such as increasing well-being, reducing physical complaints, increasing or maintaining work ability), the exact specificity of RT may seem less necessary than, e.g., in rehabilitation or high-performance sports.

It is noticeable that the majority of the studies did not specify a periodization model, which not only represents a reporting gap but also a potential for intervention optimization from the perspective of exercise science. Research shows that systematic variation in both intensity and volume leads to increased adaptation over time (83–85), which is therefore relevant in recreational or health promotion contexts. Additionally, changing one or more variables over time could have a motivating effect (86) and might have a positive influence on adherence, especially in rather less training-experienced target groups in the workplace context.

Commonly reported variables of RT exercise prescription in the included studies were muscle action, load, volume, and frequency. Therefore, in general, it was reported what was exercised, how often and with what load. Especially the determination (and continuous realization) of an appropriate load is the prerequisite for training progression. Although the load is generally specified in almost all studies (Supplementary Table 3), the exact procedures for load determination often remain rather unclear. A more precise description of the protocols or test methods used, e.g., to determine the RM or the maximal strength, would increase transparency beyond the mere mention of load and provide added value in terms of practice transfer. Moreover, with regard to training conduction, it would be critical to question the extent to which study participants are able to continuously manage the load when interventions consist of (sometimes predominantly) unsupervised training sessions (59–61, 63, 64, 67, 72, 75). As an example, the included study by Andersen et al. in which the two intervention groups performed lateral raises with elastic bands (one single-set and one multi-set group) can be referred to (59). In the associated process evaluation, 40% of participants with low adherence responded, among other things, that they felt the load progression was too fast (87). Particularly in conjunction with lower adherence, this could influence the effectiveness. For workers—some of whom may have little experience with exercise training—it may be difficult to self-direct load to, e.g., predefined repetition ranges in such a way that a constant impactful stress is ensured over the course of the intervention.

The selection of exercises was usually shown in the corresponding descriptions and illustrations, while the order of the exercises remained unclear in some studies. As complex exercises were not performed in all studies, a specific sequence, e.g., multi-joint to single-joint exercises within a session (37, 39), appears negligible in some cases, although research suggests that the exercise order should be based on priority with respect to the program goal and regardless of whether the exercise involves a relatively large or small muscle group (88). However, the intervention transparency could easily be improved by a short textual reference. Moreover, the more precise implementation (rest periods between the exercises and the sets and repetition velocity) remained partly unclear. From an exercise science perspective, rest periods in particular are important for both training planning and management (89, 90). Also, rest periods are relevant from a transfer point of view, as they determine the detailed implementation in practice, and already brief descriptions (e.g., timing or autoregulatory) would facilitate intervention replication.

In summary, reporting of RT in workplace-related interventions appears inconsistent, which is in line with reviews in, e.g., rehabilitative contexts (46–48), hindering both the replication and validation of results in follow-up studies and the implementation of successful interventions. Therefore, the use of standardized exercise-reporting tools should be further encouraged. Current guidelines from the equator network, such as the consensus on exercise reporting template (CERT) (91) or the template for intervention description and replication (TIDieR) (92), provide comprehensive guidance and were developed to address the issues outlined above. The CERT, e.g., does not only refer to the intervention itself, but also to the actual conduction (including individualization or adverse events) and implementation (including teaching/supervising expertise or setting in which exercise training is performed) (93). Thus, further important contextual factors would be captured in a more comprehensive description.

Regardless of the reporting analysis, the included studies illustrate the multifaceted nature of potential workplace-related RT interventions for adult health promotion. RT represents a promising health promotion intervention component in many respects for a wide variety of target populations (27, 33, 82, 94, 95) (for employees with, e.g., different gender, age, occupational background, or exercise experience). Most of the included studies used only small equipment such as elastic bands or dumbbells, which basically allows exercise to be performed on site (or at home) without major organizational hurdles. For interventions again with group trainings or using machines, facilities are needed to keep distances short (fitness center or training facility in the building or in close proximity). However, given the small number of exercises in most studies (Supplementary Table 3) and the small amount of time required (session duration) combined with reduced or minimal supervision, the possibility of implementing RT interventions at the workplace is generally emphasized.

### Strengths and Limitations

The main strength of the present review is the emphasis on the fundamentals of RT progression and exercise prescription (37–39). The assessment approach—oriented on a protocol by Westra et al. on the quality of RT description in COPD trials (96)—is comparatively comprehensive in terms of analyzing the reporting of RT interventions and provides a more detailed insight than, e.g., analyzing according to the so-called FITT-VP components (frequency, intensity, time, type of exercise, volume, and progression).

Since the main purpose of the review was not to investigate whether the reporting influences the intervention effects, the narrative analysis of the significant effects can be seen as a limitation. Determining the extent to which progression principles and variables of exercise prescription are attended is a critical first step in advancing knowledge of workplace-related RT interventions. Their impact on effectiveness should be explored in future analyses.

The main limitation of the review was the strict limitation to studies with a main focus on RT. During the full-text assessments, 28 studies alone were excluded that had a RT component within an intentionally mixed or multicomponent intervention. These studies would have required detailed analysis of all components (e.g., training principles for other training forms, theoretical basis for educational programs), which would have exceeded the review focus. Nevertheless, the present results should raise awareness for improved reporting not only for interventions with a main focus on RT. Future challenges will lie in the comparable reporting of training principles in more individualized or tailored approaches (33, 97) that take into account individual prerequisites and workloads in training planning and management. In addition, some studies did not definitively identify the extent to which they were workplace-related indicating the need for a better contextual description (such as location and integration into the working day).

## Conclusions

Based on the great health-promoting potential of workplace-related interventions and the often limited time available for interventions at the workplace, RT interventions in this setting should be designed to be as effective as possible. However, without comprehensive information on the actual design of workplace-related RT interventions, it remains difficult to implement optimally dosed interventions for a desired benefit in different employee target groups. Therefore, a detailed description is also relevant from a transfer perspective.

In order to increase the reproducibility of RT interventions, exercise-reporting tools should be applied more frequently (98). Furthermore, findings from exercise science should be increasingly incorporated into RT interventions in the context of workplace-related health promotion. There is still potential, especially in the integration of periodization models and the reporting of rest periods.

## Data Availability Statement

All data generated or analyzed during this study are included in the article/Supplementary Material, further inquiries can be directed to the corresponding author.

## Author Contributions

GS: conceptualization, writing—original draft, visualization, and project administration. GS and LB: methodology. GS, LB, and OM: formal analysis and investigation. AS: resources and supervision. LB, OM, and AS: writing—review and editing. All authors have read and approved the final manuscript.

## Conflict of Interest

The authors declare that the research was conducted in the absence of any commercial or financial relationships that could be construed as a potential conflict of interest.

## Publisher's Note

All claims expressed in this article are solely those of the authors and do not necessarily represent those of their affiliated organizations, or those of the publisher, the editors and the reviewers. Any product that may be evaluated in this article, or claim that may be made by its manufacturer, is not guaranteed or endorsed by the publisher.
