# Supplementary material for: Attention to Progression Principles and Variables of Exercise Prescription in Workplace-Related Resistance Training Interventions: A Systematic Review of Controlled Trials
Source: Front Public Health. 2022 Mar 25;10:832523. doi: 10.3389/fpubh.2022.832523 (PMC8990091; doi:10.3389/fpubh.2022.832523)
Supplement: Supplementary file 1 [file Table_1.DOCX]

**TABLE S1** Search terms related to workplace-related resistance training interventions for the databases LIVIVO, PubMed, SPORTDiscus, and Web of Science

| **Database** | **Search term** |
| --- | --- |
| **LIVIVO** | Open Search: worker* OR workforce OR employe* OR occupation* OR workplace OR worksite  NOT Title: diseas* OR comorbidit* OR disorder* OR patient* OR disabilit* OR rehab*  AND Open Search: (fitness OR resistance OR weight OR strength OR muscle) AND (training OR exercise OR intervention) AND ("controlled trial" OR "controlled study" OR "control group" OR RCT OR "cluster RCT")  AND Year: of 2000 until 2020 |
| **PubMed** | (worker* OR workforce OR employe* OR occupation* OR workplace OR worksite) NOT (diseas*[ti] OR comorbidit*[ti] OR disorder*[ti] OR patient*[ti] OR disabilit*[ti] OR rehab*[ti]) AND ((fitness OR resistance OR weight OR strength OR muscle) AND (training OR exercise OR intervention)) AND ("controlled trial" OR "controlled study" OR "control group" OR RCT OR "cluster RCT")  Results by year 2000 - 2020 |
| **SPORTDiscus** | ((worker* OR workforce OR employe* OR occupation* OR workplace OR worksite)) NOT TI ( diseas* OR comorbidit* OR disorder* OR patient* OR disabilit* OR rehab* ) AND (((fitness OR resistance OR weight OR strength OR muscle) AND (training OR exercise OR intervention)) AND ("controlled trial" OR "controlled study" OR "control group" OR RCT OR "cluster RCT"))  Published Date Start month: January Start Year: 2000 – End Month: December End Year: 2020 |
| **Web of Science (Core Collection, SCI-EXPANDED, SSCI)** | Topic: (worker* OR workforce OR employe* OR occupation* OR workplace OR worksite)  Not Title: (diseas* OR comorbidit* OR disorder* OR patient* OR disabilit* OR rehab*)  And Topic: (((fitness OR resistance OR weight OR strength OR muscle) AND (training OR exercise OR intervention)) AND ("controlled trial" OR "controlled study" OR "control group" OR RCT OR "cluster RCT"))  Publication Date 2000-01-01 to 2020-12-31 |
