# Supplementary material for: Attention to Progression Principles and Variables of Exercise Prescription in Workplace-Related Resistance Training Interventions: A Systematic Review of Controlled Trials
Source: Front Public Health. 2022 Mar 25;10:832523. doi: 10.3389/fpubh.2022.832523 (PMC8990091; doi:10.3389/fpubh.2022.832523)
Supplement: Supplementary file 2 [file Table_2.DOCX]

All included studies were subjected to the Effective Public Health Practice Project`s (EPHPP) quality assessment tool for quantitative studies (1, 2). According to the EPHPP dictionary (3), the first six components were included in the assessment (selection bias, study design, confounders, blinding, data collection methods, and withdrawals and dropouts) and rated as weak, moderate or strong.

**TABLE S2** Assessment of study quality using the Effective Public Health Practice Project`s (EPHPP) quality assessment tool

| **Reference** | **Selection bias** | **Study design** | **Confounders** | **Blinding** | **Data collection methods** | **Withdrawals and dropouts** | **Global rating** |
| --- | --- | --- | --- | --- | --- | --- | --- |
| Andersen et al. (2008) (4) | Weak | Strong | Strong | Weak | Strong | Weak | Weak |
| Andersen et al. (2011) (5) | Weak | Strong | Strong | Moderate | Strong | Strong | Moderate |
| Andersen et al. (2012) (6) | Moderate | Strong | Strong | Weak | Strong | Weak | Weak |
| Blangsted et al. (2008) (7) | Weak | Strong | Strong | Weak | Strong | Strong | Weak |
| Escriche-Escuder et al. (2020) (8) | Strong | Moderate | Weak | Moderate | Strong | Strong | Moderate |
| Gram et al. (2014) (9) | Moderate | Strong | Strong | Weak | Strong | Weak | Weak |
| Haufe et al. (2017) (10) | Weak | Strong | Strong | Strong | Strong | Strong | Moderate |
| Helmhout et al. (2004) (11) | Moderate | Strong | Weak | Moderate | Strong | Moderate | Moderate |
| Helmhout et al. (2008) (12) | Moderate | Strong | Weak | Moderate | Strong | Strong | Moderate |
| Li et al. (2017) (13) | Moderate | Strong | Strong | Moderate | Strong | Strong | Strong |
| Mayer et al. (2015) (14) | Weak | Strong | Strong | Moderate | Strong | Strong | Moderate |
| Mulla et al. (2018) (15) | Moderate | Strong | Weak | Moderate | Strong | Strong | Moderate |
| Muñoz-Poblete et al. (2019) (16) | Moderate | Strong | Strong | Moderate | Strong | Weak | Moderate |
| Nygaard Andersen et al. (2017) (17) | Weak | Strong | Weak | Weak | Strong | Strong | Weak |
| Sjögren et al. (2006) (18) | Moderate | Strong | Strong | Moderate | Strong | Strong | Strong |
| Sundstrup et al (2014) (19) | Moderate | Strong | Strong | Moderate | Strong | Strong | Strong |
| Zavanela et al. (2012) (20) | Moderate | Strong | Weak | Weak | Strong | Weak | Weak |
| Zebis et al. (2011) (21) | Moderate | Strong | Strong | Weak | Strong | Strong | Moderate |

**References**

1. Effective Public Health Practice Project. *Quality Assessment Tool for Quantitative Studies* [cited 2021 Sep 30]. Available from: https://www.ephpp.ca/PDF/Quality%20Assessment%20Tool_2010_2.pdf

2. Thomas BH, Ciliska D, Dobbins M, Micucci S. A process for systematically reviewing the literature: providing the research evidence for public health nursing interventions. *Worldviews on Evidence-Based Nursing* (2004) **1**:176–84. doi:10.1111/j.1524-475X.2004.04006.x

3. Effective Public Health Practice Project. *Quality Assessment Tool for Quantitative Studies Dictionary* [cited 2021 Sep 30]. Available from: https://www.ephpp.ca/PDF/QADictionary_dec2009.pdf

4. Andersen LL, Kjaer M, Søgaard K, Hansen L, Kryger AI, Sjøgaard G. Effect of two contrasting types of physical exercise on chronic neck muscle pain. *Arthritis Rheum* (2008) **59**:84–91. doi:10.1002/art.23256

5. Andersen LL, Saervoll CA, Mortensen OS, Poulsen OM, Hannerz H, Zebis MK. Effectiveness of small daily amounts of progressive resistance training for frequent neck/shoulder pain: randomised controlled trial. *Pain* (2011) **152**:440–6. doi:10.1016/j.pain.2010.11.016

6. Andersen CH, Andersen LL, Gram B, Pedersen MT, Mortensen OS, Zebis MK, et al. Influence of frequency and duration of strength training for effective management of neck and shoulder pain: a randomised controlled trial. *Br J Sports Med* (2012) **46**:1004–10. doi:10.1136/bjsports-2011-090813

7. Blangsted AK, Søgaard K, Hansen EA, Hannerz H, Sjøgaard G. One-year randomized controlled trial with different physical-activity programs to reduce musculoskeletal symptoms in the neck and shoulders among office workers. *Scand J Work Environ Health* (2008) **34**:55–65. doi:10.5271/sjweh.1192

8. Escriche-Escuder A, Calatayud J, Andersen LL, Ezzatvar Y, Aiguadé R, Casaña J. Effect of a brief progressive resistance training program in hospital porters on pain, work ability, and physical function. *Musculoskelet Sci Pract* (2020) **48**:102162. doi:10.1016/j.msksp.2020.102162

9. Gram B, Andersen C, Zebis MK, Bredahl T, Pedersen MT, Mortensen OS, et al. Effect of training supervision on effectiveness of strength training for reducing neck/shoulder pain and headache in office workers: cluster randomized controlled trial. *Biomed Res Int* (2014) **2014**:693013. doi:10.1155/2014/693013

10. Haufe S, Wiechmann K, Stein L, Kück M, Smith A, Meineke S, et al. Low-dose, non-supervised, health insurance initiated exercise for the treatment and prevention of chronic low back pain in employees. Results from a randomized controlled trial. *PLoS ONE* (2017) **12**:e0178585. doi:10.1371/journal.pone.0178585

11. Helmhout PH, Harts CC, Staal JB, Candel MJ, Bie RA de. Comparison of a high-intensity and a low-intensity lumbar extensor training program as minimal intervention treatment in low back pain: a randomized trial. *Eur Spine J* (2004) **13**:537–47. doi:10.1007/s00586-004-0671-y

12. Helmhout PH, Harts CC, Viechtbauer W, Staal JB, Bie RA de. Isolated lumbar extensor strengthening versus regular physical therapy in an army working population with nonacute low back pain: a randomized controlled trial. *Arch Phys Med Rehabil* (2008) **89**:1675–85. doi:10.1016/j.apmr.2007.12.050

13. Li X, Lin C, Liu C, Ke S, Wan Q, Luo H, et al. Comparison of the effectiveness of resistance training in women with chronic computer-related neck pain: a randomized controlled study. *Int Arch Occup Environ Health* (2017) **90**:673–83. doi:10.1007/s00420-017-1230-2

14. Mayer JM, Quillen WS, Verna JL, Chen R, Lunseth P, Dagenais S. Impact of a supervised worksite exercise program on back and core muscular endurance in firefighters. *Am J Health Promot* (2015) **29**:165–72. doi:10.4278/ajhp.130228-QUAN-89

15. Mulla DM, Wiebenga EG, Chopp-Hurley JN, Kaip L, Jarvis RS, Stephens A, et al. The Effects of Lower Extremity Strengthening Delivered in the Workplace on Physical Function and Work-Related Outcomes Among Desk-Based Workers: A Randomized Controlled Trial. *J Occup Environ Med* (2018) **60**:1005–14. doi:10.1097/JOM.0000000000001408

16. Muñoz-Poblete C, Bascour-Sandoval C, Inostroza-Quiroz J, Solano-López R, Soto-Rodríguez F. Effectiveness of Workplace-Based Muscle Resistance Training Exercise Program in Preventing Musculoskeletal Dysfunction of the Upper Limbs in Manufacturing Workers. *J Occup Rehabil* (2019) **29**:810–21. doi:10.1007/s10926-019-09840-7

17. Nygaard Andersen L, Mann S, Juul-Kristensen B, Søgaard K. Comparing the Impact of Specific Strength Training vs General Fitness Training on Professional Symphony Orchestra Musicians: A Feasibility Study. *Med Probl Perform Art* (2017) **32**:94–100. doi:10.21091/mppa.2017.2016

18. Sjögren T, Nissinen KJ, Järvenpää SK, Ojanen MT, Vanharanta H, Mälkiä EA. Effects of a physical exercise intervention on subjective physical well-being, psychosocial functioning and general well-being among office workers: a cluster randomized-controlled cross-over design. *Scand J Med Sci Sports* (2006) **16**:381–90. doi:10.1111/j.1600-0838.2005.00516.x

19. Sundstrup E, Jakobsen MD, Andersen CH, Jay K, Persson R, Aagaard P, et al. Effect of two contrasting interventions on upper limb chronic pain and disability: a randomized controlled trial. *Pain Physician* (2014) **17**:145–54.

20. Zavanela PM, Crewther BT, Lodo L, Florindo AA, Miyabara EH, Aoki MS. Health and fitness benefits of a resistance training intervention performed in the workplace. *J Strength Cond Res* (2012) **26**:811–7. doi:10.1519/JSC.0b013e318225ff4d

21. Zebis MK, Andersen LL, Pedersen MT, Mortensen P, Andersen CH, Pedersen MM, et al. Implementation of neck/shoulder exercises for pain relief among industrial workers: a randomized controlled trial. *BMC Musculoskelet Disord* (2011) **12**:205. doi:10.1186/1471-2474-12-205
