# Supplementary material for: Attention to Progression Principles and Variables of Exercise Prescription in Workplace-Related Resistance Training Interventions: A Systematic Review of Controlled Trials
Source: Front Public Health. 2022 Mar 25;10:832523. doi: 10.3389/fpubh.2022.832523 (PMC8990091; doi:10.3389/fpubh.2022.832523)
Supplement: Supplementary file 3 [file Table_3.DOCX]

Principles of RT progression (1, 2) and variables of RT exercise prescription (1–3) were extracted from the methods sections of the included articles or additionally from the underlying study protocols, respectively, and rated independently according to the working understandings. Each principle and each variable was rated as follows: yes (+) “reported/applied”, no (-) “not reported/not applied”, or unclear (?) if it was unclear or inconsistent whether a principle/variable was reported/applied. “Not applicable” (na) was recorded for “exercise order” and “rest between exercises” if only one exercise was used in the intervention and for “rest between sets” if only single sets were used.

**TABLE S3** Application of principles of progression and variables of exercise prescription (citations or text passages)

| **#** | **Reference** | **Principle** | | | **Sum score^1^** | **Variable** | | | | | | | | | **Sum score^2^** |
| --- | --- | --- | --- | --- | --- | --- | --- | --- | --- | --- | --- | --- | --- | --- | --- |
|  |  | **Progressive overload** | **Specificity** | **Periodization** |  | **Muscle action** | **Loading** | **Volume** | **Exercise selection** | **Exercise order** | **Rest periods** | | **Velocity** | **Frequency** |  |
|  |  |  |  |  |  |  |  |  |  |  | Between sets | Between exercises |  |  |  |
| **1** | Andersen et al. (2008) (4) | **(+)**: “During the intervention period the training load was progressively increased according to the principle of periodization and progressive overload” | **(+)**: “specific strength training locally for the neck and shoulder muscles” | **(+)**: “relative loadings were progressively increased from 12 repetitions maximum (RM; ~70% of maximal intensity) at the beginning of the training period to 8 RM (~80% of maximal intensity) during the later phase” (classical model) | **3/3** | **(+)**: “exercises were performed in a conventional manner using consecutive concentric and eccentric muscle contractions” | **(+)**:“12 repetitions maximum (RM; ~70% of maximal intensity)” / “8 RM (~80% of maximal intensity)” | **(+)** number of repetitions: “12 repetitions maximum” / “8 RM”, number of sets: “3 sets per exercise”, number of exercises: “Three (…) exercises (…) during each training session” | **(+)**:”5 different dumbbell exercises (1-arm row, shoulder abduction, shoulder elevation, reverse flies, and upright row).” | **(+)**: ”Three of the 5 different exercises (…) were performed during each training session in an alternating manner, with shoulder elevation being the only exercise that was performed during each session.“ | **(-)**: not specified | **(-)**: not specified | **(?)**: “each set typically lasted 25–35 seconds“ (relationship between the concentric and eccentric phases unclear) | **(+)**: “training was performed (…) 3 times per week” | **6/9** |
| **2** | Andersen et al. (2011) (5) | **(+)**: “progressive resistance training” | **(+)**: “shoulder abductions (…) for effectively targeting several relevant neck/shoulder muscles” | **(-)**: not specified/applied | **2/3** | **(+)**: “raise both arms slightly in front of the body (…). (…) entire range of motion.” | **(+)**: “During the first 2 weeks (…) moderate resistance (…). After 2 weeks (…) instructions to increase resistance again when they could complete 6 sets of 12 repetitions” / “only a single set to failure“. | **(+)** number of repetitions: “8 to 12” / “single set to failure”, number of sets: “5 to 6” / “single set”, number of exercises: 1 | **(+)**: “lateral raise” | **(na):** only 1 exercise | **(+):** “new sets every other minute” | **(na):** only 1 exercise | **(+)**: “raising and lowering the arms in approximately 2 s” | **(+)**: “5 times per week” | **7/7** |
| **3** | Andersen et al. (2012) (6)  (protocol: (7)) | **(+)**: “training loads were progressively Increased“ | **(+)** protocol: “specific strength training (…), as shown in figure 2, for the neck and shoulder muscles” | **(+)** protocol: “The first 12 weeks of the program followed the principles of linear periodization and the last 8 weeks the principles of undulating periodization” (classical/undulating model) | **3/3** | **(+)**: dynamic execution of the exercises described in the protocol | **(+)** protocol: “20 repetitions maximum (RM) at the beginning of the intervention-period to 8 RM during the later phase” | **(?)** protocol: number of repetitions: see table 1, number of sets: total number see table 1 (sets per exercise unclear), number of exercises: “The four training-groups performed the same total amount of exercises (…) per week” (exercises per week unclear) | **(+)**: “five different dumbbell exercises: front raise, lateral raise, reverse flies, shrugs and wrist extension” | **(+)** protocol: “Participants performed exercises in a rotating manner” | **(+)** protocol: “1-2 minutes” | **(-)**: unclear/not specified, as it is also not clear whether several exercises were performed per session depending on the intervention group (protocol: “respective exercise of that day”) | **(-)**: not specified | **(+)**: “once a week” / “three times a week” / ”nine times a week | **6/9** |
| **4** | Blangsted et al. (2008) (8) | **(+)**: “encouraged to add to the load” | **(+)**: “exercises specifically for the muscles in the shoulder and neck region” | **(-)**: not specified/applied | **2/3** | **(+)**: “dynamic (…), static (…), and explosive” | **(+)**: “~10–15 repetition maximum“ (dynamic exercises) | **(+)** number of repetitions: “10-15”, number of sets: “2-3”, number of exercises: 4 (dynamic exercises) | **(+)**: “training was performed with dumbbells and consisted of the following exercises: shoulder extension, shoulder abduction, shoulder abduction with special attention to the supraspinatus muscle, and shoulder lift” | **(?)**: not explicitly clear whether the listing was also the (fixed) order | **(-)**: not specified | **(-)**: not specified | **(-)**: not specified (dynamic exercises) | **(+)**: “three sessions per week” | **5/9** |
| **5** | Escriche-Escuder et al. (2020) (9) | **(+)**: “Intensity progressively increased during the program” | **(?)**: general health-promoting whole body training without a "specific" focus | **(+)**: “intensity progressed from 20 repetitions maximum (RM) at the beginning of the training period to 8 RM during the later phase” (classical model) | **2/3** | **(+)**: “5 dynamic exercises (...). (...) exercise 6 was performed isometrically” | **(+)**: “20 repetitions maximum (RM)” / ”8 RM” (exercises 1-4) / “to failure” (exercise 5) / “modifying the complexity” (exercise 6) | **(+)** number of repetitions: 20, 15, 12, 10, 8 (Table 1), number of sets: 1 (Table 1), number of exercises: “6 resistance exercises” | **(+)**: “(1) squat, (2) back squeeze, (3) deadlifts, (4) torso-twist, (5) push-ups, (6) side bridge” | **(+)**: “The order of execution of the exercises was always the same, except for the first exercise which changed with each training progression.” (see further description) | **(na)**: only 1 set per exercise | **(+)**: “exercises were performed in circuit, quickly changing from an exercise to the next without break” | **(+)**: “consecutive concentric (2s) and eccentric (2s) muscle contractions” | **(+)**: “five sessions per week” | **8/8** |
| **6** | Gram et al. (2014) (10)  (protocol: (7), same as (6)) | **(+)**: “training intensity and the loads were progressively increased” | **(+)** protocol: “specific strength training (…), as shown in figure 2, for the neck and shoulder muscles” | **(+)** protocol: “The first 12 weeks of the program followed the principles of linear periodization and the last 8 weeks the principles of undulating periodization” (classical/undulating model) | **3/3** | **(+)**: dynamic execution of the exercises described in the protocol | **(+)** protocol: “20 repetitions maximum (RM) at the beginning of the intervention-period to 8 RM during the later phase” | **(?)** protocol: number of repetitions: see table 1, number of sets: total number see table 1 (sets per exercise unclear), number of exercises: “The four training-groups performed the same total amount of exercises (…) per week” (exercises per week unclear) | **(+)** protocol: “A) Front raise, B) lateral raise, C) reverse flies, D) shrugs, and E) wrist extension” (figure 2) | **(+)** protocol: “Participants performed exercises in a rotating manner” | **(+)** protocol: “1-2 minutes” | **(-)**: number of exercises per session unclear (protocol: “respective exercise of that day”) | **(-)**: not specified | **(+)**: “three times per week” | **6/9** |
| **7** | Haufe et al. (2017) (11) | **(-)**: not mentioned | **(+)**: “Training included generally established exercises for the trunk musculature particularly for the lower back including exercises for extensor muscles of the lower spine, abdominal muscles, muscles involved in lateral trunk flexion and muscles of the rotator cuff of the shoulder.” | **(-)**: not specified/applied | **1/3** | **(+)**: “concentric, eccentric or isometric muscle actions” | **(?)**: “During the regular counsellings the physiotherapist (…) adapted the exercises in regard to the sets, repetitions and intensity of training at its own discretion” | **(?)**: number of repetitions: exercise specific given in the supplementary material, number of sets: exercise specific given in the supplementary material, number of exercises: unclear (compare right) | **(?)**: “subjects received comprehensive instructions and demonstrations of four to six exercises chosen from a list (see S1 Dataset for the list of exercises (…))” | **(-)**: ambiguities regarding the order due to the textual description ( compare left) and the listing in the supplementary material | **(-)**: not specified | **(-)**: not specified | **(?)**: “moderate contraction velocity“ | **(+)**: “three times per week” | **2/9** |
| **8** | Helmhout et al. (2004) (12) | **(+)**: “progressive resistance training“ | **(+)**: “isolated lumbar extensor muscle groups” | **(?)**: probably the change in the number of repetitions from week 2 to week 3 in the 12-week intervention does not follow a systematic periodization | **2/3** | **(+)**: “flexion and extension” | **(+)**: “15 to 20 repetitions (…) or 10 to 15 repetitions (…), equivalent to approx. 50% and 70% of the one-repetition maximum (1 RM), respectively” | **(+)**: number of repetitions: “15 to 20” / “10 to 15”, number of sets: 1, number of exercises: 1 | **(+)**: “standard lower back machine” | **(na)**: only 1 exercise | **(na)**: only 1 set | **(na)**: only 1 exercise | **(+)**: “moving in 2 s from maximal flexion to maximal extension (…), and returning from maximal extension to maximal flexion in 4 s” | **(+)**: “2 days/week“ / “1 day/week” | **6/6** |
| **9** | Helmhout et al. (2008) (13) (protocol: (14)) | **(+)**: “progressive resistance training (...). (...) If the subject was able to perform more than 20 repetitions (...), 2.5kg was added in the next training session” | **(+)**: “lumbar extensor strength training” | **(-)**: not specified/applied | **2/3** | **(+)**: “extension (...) and flexion” | **(+)**: “15 to 20 repetitions (...) on the lower back machine, which is equivalent to approximately 50% to 70 % of the 1-RM, respectively” | **(+)**: number of repetitions: “15 to 20”, number of sets: 1, number of exercises: 1 | **(+)**: “Total Trunk Rehab machine. (...) lower back machine” | **(na)**: only 1 exercise | **(na)**: only 1 set | **(na)**: only 1 exercise | **(+)**: “slow and controlled back extension (2s) and flexion (4s) movements” | **(+)**: “twice a week” | **6/6** |
| **10** | Li et al. (2017) (15) | **(+)**: „progressive resistance training“ | **(+)**: “cervical (…) exercises” | **(-)**: not specified/applied | **2/3** | **(+)**: “isometric” | **(+)**: “30% (…) maximal strength (…) and then increased to 50 and 70%” | **(+)**: number of repetitions: “8-12”, number of sets: presumably only one set per exercise, number of exercises: 5 (“training session (...) each exercise”) | **(+)**: “exercises (flexion, extension, left lateral flexion, and right lateral flexion)“ | **(?)**: not explicitly clear whether the listing was also the (fixed) order | **(na)** presumably only one set per exercise | **(-)**: not specified | **(+)**: “repetitions of 5 s duration“ | **(+)**: “at least three times a week” | **6/8** |
| **11** | Mayer et al. (2015) (16) | **(+)**: “progressive resistance exercise“ | **(+)**: “exercise protocol was based on prior literature suggesting its effectiveness in activating the back and core muscles and improving back and core muscle endurance“ | **(-)**: not specified/applied | **2/3** | **(+)**: “isometric” / “dynamic” | **(+)**: “One set of five repetitions was completed for each exercise“ (mat-based exercises) / “performed until volitional fatigue or 30 repetitions was attained” (back extension” | **(+)**: number of repetitions: “five repetitions” (mat-based exercises) / “until volitional fatigue or 30 repetitions“ (back extensions), number of sets: one set per exercise, number of exercises: 5 | **(+)**: “four mat-based core exercises (cat camel, birddog, curl-up, and side bridge) (…). (…) Following the core exercises, (…) back extension on a variable angle Roman chair” | **(?)**: not explicitly clear whether the listing was also the (fixed) order | **(na)**: only 1 set per exercise | **(+)**: “no rest between exercises“ | **(+)**: “isometric contraction lasting 6 to 8 seconds“ / “eccentric phase (…) 4 seconds. (…) concentric phase of exercise, (… ) 4 seconds. (…) held the terminal extension position for 4 seconds” | **(+):** “2 days per week“ | **7/8** |
| **12** | Mulla et al. (2018) (17) | **(+)**: “Exercises progressed (...) throughout the 12-week period” | **(?)**: unclear why the osteoarthritis training/leg-strengthening classes described would be "specific" for the group of desk-based workers | **(?)**: “increasing the number and duration of repetitions” (volume) but not e.g. decreasing the intensity | **1/3** | **(+)**: “static squats and lunges as well as other exercises” | **(+)**: “rating of perceived exertion (RPE) between 5 and 7 from a scale of 0 to 10” | **(-)**: number of repetitions: not to be determined, number of sets: not to be determined, number of exercises: not to be determined | **(?)**: “squats and lunges as well as other exercises” | **(-)**:not to be determined | **(-)**:not to be determined | **(-)**:not to be determined | **(-)**: not specified | **(+)**: “three (...) classes per week” | **3/9** |
| **13** | Muñoz-Poblete et al. (2019) (18) | **(+)**: “muscle training with progressive resistance” | **(+)**: “Training (...) focusing on three areas of the body: scapular waist zone (...), shoulder zone (...), forearm-hand zone” | **(-)**: not specified/applied | **2/3** | **(+)**: “concentric (...), isometric (..,) and eccentric” | **(+)**: “program was standardized (...) making only differences between men and women. (...) 4.6 kg, 6.3 kg, 8.5 kg (...). (...) 3.2 kg, 4.6 kg, 6.3 kg” | **(-)**: number of repetitions: not to be determined, number of sets: not to be determined, number of exercises: not to be determined | **(+)**: “Fig. 2 1. Elevator muscles; 2. Retractor muscles; 3. Protractor muscles; 4. Abductor muscles; 5. Rotator muscles; 6. Supinator muscles; and 7. Extensor muscles“ | **(?)**: minor ambiguities between textual description and indicated numbering in the figure | **(-)** “not clear how many sets or cycles were finally carried out” | **(?)**: unclear what the stated time of rest refers to (“The pause between each cycle of exercises was 10 s”) | **(+)**: “concentric contraction, isometric contraction for 6 s, and finally eccentric contraction” | **(+)**: “three times per week” | **5/9** |
| **14** | Nygaard Andersen et al. (2017) (19) | **(+)**: “During the intervention period, the training load in a set was progressively increased” | **(+)**: “supervised high-intensity specific strength training, focusing on the neck and shoulder muscles | **(+)**: classical model (compare “Load” on the right) | **3/3** | **(+)**: “consecutive concentric and eccentric muscle contractions“ | **(+)**: “load was individually assessed and adjusted so it corresponded to the maximum load that could be lifted 15 times (15 repetitions maximum ~70% of maximal intensity). During the intervention period, the training load (…) increased from the 15 repetitions maximum at the beginning of the training period to 8–12 repetitions maximum (~75–85% of maximal intensity)” | **(+)**: number of repetitions: “15 repetitions maximum at the beginning (…) to 8–12 repetitions maximum (…) during the later phase”, number of sets: ”three sets per exercise”, number of exercises: “each training session, three (...) exercises” | **(+)**: “five dumbbell exercises; one-arm row (…), shoulder abduction (…), shoulder elevation (…), reverse flies (…), and upright row” | **(+)**: “During each training session, three of the five exercises were performed (…) following an undulating schedule, shoulder elevation being the only exercise that was performed during each session” | **(-)**: not specified | **(-)**: not specified | **(-)**: not specified | **(+)** “3 times/week” | **6/9** |
| **15** | Sjögren et al. (2006) (20) | **(+)**: “progressive (…) resistance training“ | **(?)**:general health-promoting training without a "specific" focus | **(-)**: not specified/applied | **1/3** | **(+)**: “dynamic” | **(+)**: “participants’ perceptions of exertion (Borg RPE 6–20) during light resistance training to set the training load to 30% of 1 RM” | **(+)**: number of repetitions: “20 times”, number of sets: presumably only one set per exercise, number of exercises: 6 | **(+)**: “six (…) movements: upper extremity extension, upper extremity flexion, trunk rotation to the right, trunk rotation to the left, knee extension and knee flexion” | **(+)**: “There was no defined sequence between the training movements, except that the physiotherapist recommended that, in the case of the upper extremity movements, flexion should be performed after extension” | **(na)** presumably only one set per exercise | **(+)**: “30 s pause between the training movements“ | **(+)**: “single repetition of a training movement was 1.8 s” | **(+)**: “five times a week” / “seven to eight times a week” | **8/8** |
| **16** | Sundstrup et al (2014) (21) (protocol (22) | **(+)**: “Training intensity (loads) was progressively increased“ | **(+)**: “resistance training for the shoulder, arm, and hand muscles“ | **(+)**: “Training intensity (loads) was progressively increased from 20 repetition maximum (RM) at the beginning of the training period to 8 RM during the latter phase” (classical model) | **3/3** | **(+)** protocol: “The exercises (…) were performed in a conventional manner using consecutive concentric and eccentric muscle contractions (…). (…) twist performed (…) eccentrically.” | **(+)** protocol: “20 repetitions maximum (RM)” / “8 RM” / “20-30 RM” | **(+)** protocol: number of repetitions: “20 repetitions maximum (RM)” / “8 RM” / “20-30 RM”, number of sets: “3 sets per exercise”, number of exercises: “3-4 (…) exercises” | **(+)**: “8 resistance exercises: 1 – 2: shoulder rotation (…), 3 – 4: ulnar and radial deviation of the wrist (…), 5: eccentric training of the wrist extensors (…), 6: wrist flexion and extension (…), 7: flexion of the hand (…), 8: extension of the hand and fingers” | **(+)** protocol: “3–4 of the 8 different exercises (…) during each training session in an alternating manner” | **(-)**: not specified | **(-)**: not specified | **(?)** protocol: “in a controlled manner” | **(+)**: “3 times a week” | **6/9** |
| **17** | Zavanela et al. (2012) (23) | **(+)**: “The training stimulus was also progressed by increasing the absolute load lifted for each exercise over time” | **(?)**: general health-promoting whole body training without a "specific" focus | **(-)**: not specified/applied | **1/3** | **(?)**: implicitly the exercises should be dynamic | **(+)**: “individual loading parameters based on their repetition maximum (RM) lifts (…). (…)10–12 repetitions with a 10–12RM load for each exercise” | **(+)**: number of repetitions: “10-12 repetitions”, number of sets: “3 sets”, number of exercises: see table 1 | **(+)**: “full body program (…) performed from weeks 1 to 8 using 12 exercises (…) (Table 1). A split body program (…) from weeks 9 to 24 (see Table 1 for exercises)” | **(?)**: not explicitly clear whether the table listing was also the (fixed) order | **(+)**: “1-minute rest periods between sets” | **(+)**: “1-minute rest periods between (…) exercises” | **(-)**: not specified | **(+)**: “3 training sessions from weeks 0 to 8 and 4 training sessions from weeks 9 to 24” | **6/9** |
| **18** | Zebis et al. (2011) (24) | **(+)**: “training load was progressively increased“ | **(+)**: “locally for the neck and shoulder muscles” | **(+)**: “both linear (…) and undulating periodization“(classical/undulating model) | **3/3** | **(+)**: “consecutive concentric and eccentric muscle contractions” | **(+)**: “15 repetitions maximum (RM; ~70% of maximal intensity) at the beginning of the training period to 8-12 RM (~75-85% of maximal intensity) during the later phase“ | **(?)**: number of repetitions: “15 repetitions (…) at the beginning (…) to 8-12 RM (…) during the later phase”, number of sets: total number see figure 3 (sets per exercise unclear), number of exercises: 5 | **(+):** “five training exercises used in the present study. A) Front raise, B) lateral raise, C) reverse flies, D) shrugs, and E) wrist extension“ (figure 2) | **(?)**: not explicitly clear whether the listing was also the (fixed) order | **(-)**: not specified | **(-)**: not specified | **(?)**: “slow to moderate (...) velocity” | **(+)**: “three sessions per week” | **4/9** |
| **Proportion** | | 94 % (17/18) | 78 % (14/18) | 39 % (7/18) | **70 % (38/54)** | 94 % (17/18) | 94 % (17/18) | 67 % (12/18) | 89 % (16/18) | 47 % (7/15) | 33 % (4/12) | 27 % (4/15) | 44 % (8/18) | 100 % (18/18) | **69 % (103/150)** |

^1^Possible maximum score = 3. ^2^Possible maximum score = 9 (depending on the correction for the number of “na”).

**References**

1. Ratamess NA, Alvar BA, Evetoch TK, Housh TJ, Kibler WB, Kraemer WJ, et al. American College of Sports Medicine position stand. Progression models in resistance training for healthy adults. *Med Sci Sports Exerc* (2009) **41**:687–708. doi:10.1249/MSS.0b013e3181915670

2. Kraemer WJ, Ratamess NA. Fundamentals of resistance training: progression and exercise prescription. *Med Sci Sports Exerc* (2004) **36**:674–88. doi:10.1249/01.MSS.0000121945.36635.61

3. Bird SP, Tarpenning KM, Marino FE. Designing resistance training programmes to enhance muscular fitness: a review of the acute programme variables. *Sports Med* (2005) **35**:841–51. doi:10.2165/00007256-200535100-00002

4. Andersen LL, Kjaer M, Søgaard K, Hansen L, Kryger AI, Sjøgaard G. Effect of two contrasting types of physical exercise on chronic neck muscle pain. *Arthritis Rheum* (2008) **59**:84–91. doi:10.1002/art.23256

5. Andersen LL, Saervoll CA, Mortensen OS, Poulsen OM, Hannerz H, Zebis MK. Effectiveness of small daily amounts of progressive resistance training for frequent neck/shoulder pain: randomised controlled trial. *Pain* (2011) **152**:440–6. doi:10.1016/j.pain.2010.11.016

6. Andersen CH, Andersen LL, Gram B, Pedersen MT, Mortensen OS, Zebis MK, et al. Influence of frequency and duration of strength training for effective management of neck and shoulder pain: a randomised controlled trial. *Br J Sports Med* (2012) **46**:1004–10. doi:10.1136/bjsports-2011-090813

7. Andersen LL, Zebis MK, Pedersen MT, Roessler KK, Andersen CH, Pedersen MM, et al. Protocol for work place adjusted intelligent physical exercise reducing musculoskeletal pain in shoulder and neck (VIMS): a cluster randomized controlled trial. *BMC Musculoskelet Disord* (2010) **11**:173. doi:10.1186/1471-2474-11-173

8. Blangsted AK, Søgaard K, Hansen EA, Hannerz H, Sjøgaard G. One-year randomized controlled trial with different physical-activity programs to reduce musculoskeletal symptoms in the neck and shoulders among office workers. *Scand J Work Environ Health* (2008) **34**:55–65. doi:10.5271/sjweh.1192

9. Escriche-Escuder A, Calatayud J, Andersen LL, Ezzatvar Y, Aiguadé R, Casaña J. Effect of a brief progressive resistance training program in hospital porters on pain, work ability, and physical function. *Musculoskelet Sci Pract* (2020) **48**:102162. doi:10.1016/j.msksp.2020.102162

10. Gram B, Andersen C, Zebis MK, Bredahl T, Pedersen MT, Mortensen OS, et al. Effect of training supervision on effectiveness of strength training for reducing neck/shoulder pain and headache in office workers: cluster randomized controlled trial. *Biomed Res Int* (2014) **2014**:693013. doi:10.1155/2014/693013

11. Haufe S, Wiechmann K, Stein L, Kück M, Smith A, Meineke S, et al. Low-dose, non-supervised, health insurance initiated exercise for the treatment and prevention of chronic low back pain in employees. Results from a randomized controlled trial. *PLoS ONE* (2017) **12**:e0178585. doi:10.1371/journal.pone.0178585

12. Helmhout PH, Harts CC, Staal JB, Candel MJ, Bie RA de. Comparison of a high-intensity and a low-intensity lumbar extensor training program as minimal intervention treatment in low back pain: a randomized trial. *Eur Spine J* (2004) **13**:537–47. doi:10.1007/s00586-004-0671-y

13. Helmhout PH, Harts CC, Viechtbauer W, Staal JB, Bie RA de. Isolated lumbar extensor strengthening versus regular physical therapy in an army working population with nonacute low back pain: a randomized controlled trial. *Arch Phys Med Rehabil* (2008) **89**:1675–85. doi:10.1016/j.apmr.2007.12.050

14. Helmhout PH, Harts CC, Staal JB, Bie RA de. Rationale and design of a multicenter randomized controlled trial on a 'minimal intervention' in Dutch army personnel with nonspecific low back pain [ISRCTN19334317]. *BMC Musculoskelet Disord* (2004) **5**:40. doi:10.1186/1471-2474-5-40

15. Li X, Lin C, Liu C, Ke S, Wan Q, Luo H, et al. Comparison of the effectiveness of resistance training in women with chronic computer-related neck pain: a randomized controlled study. *Int Arch Occup Environ Health* (2017) **90**:673–83. doi:10.1007/s00420-017-1230-2

16. Mayer JM, Quillen WS, Verna JL, Chen R, Lunseth P, Dagenais S. Impact of a supervised worksite exercise program on back and core muscular endurance in firefighters. *Am J Health Promot* (2015) **29**:165–72. doi:10.4278/ajhp.130228-QUAN-89

17. Mulla DM, Wiebenga EG, Chopp-Hurley JN, Kaip L, Jarvis RS, Stephens A, et al. The Effects of Lower Extremity Strengthening Delivered in the Workplace on Physical Function and Work-Related Outcomes Among Desk-Based Workers: A Randomized Controlled Trial. *J Occup Environ Med* (2018) **60**:1005–14. doi:10.1097/JOM.0000000000001408

18. Muñoz-Poblete C, Bascour-Sandoval C, Inostroza-Quiroz J, Solano-López R, Soto-Rodríguez F. Effectiveness of Workplace-Based Muscle Resistance Training Exercise Program in Preventing Musculoskeletal Dysfunction of the Upper Limbs in Manufacturing Workers. *J Occup Rehabil* (2019) **29**:810–21. doi:10.1007/s10926-019-09840-7

19. Nygaard Andersen L, Mann S, Juul-Kristensen B, Søgaard K. Comparing the Impact of Specific Strength Training vs General Fitness Training on Professional Symphony Orchestra Musicians: A Feasibility Study. *Med Probl Perform Art* (2017) **32**:94–100. doi:10.21091/mppa.2017.2016

20. Sjögren T, Nissinen KJ, Järvenpää SK, Ojanen MT, Vanharanta H, Mälkiä EA. Effects of a physical exercise intervention on subjective physical well-being, psychosocial functioning and general well-being among office workers: a cluster randomized-controlled cross-over design. *Scand J Med Sci Sports* (2006) **16**:381–90. doi:10.1111/j.1600-0838.2005.00516.x

21. Sundstrup E, Jakobsen MD, Andersen CH, Jay K, Persson R, Aagaard P, et al. Effect of two contrasting interventions on upper limb chronic pain and disability: a randomized controlled trial. *Pain Physician* (2014) **17**:145–54.

22. Sundstrup E, Jakobsen MD, Andersen CH, Jay K, Persson R, Aagaard P, et al. Participatory ergonomic intervention versus strength training on chronic pain and work disability in slaughterhouse workers: study protocol for a single-blind, randomized controlled trial. *BMC Musculoskelet Disord* (2013) **14**:67. doi:10.1186/1471-2474-14-67

23. Zavanela PM, Crewther BT, Lodo L, Florindo AA, Miyabara EH, Aoki MS. Health and fitness benefits of a resistance training intervention performed in the workplace. *J Strength Cond Res* (2012) **26**:811–7. doi:10.1519/JSC.0b013e318225ff4d

24. Zebis MK, Andersen LL, Pedersen MT, Mortensen P, Andersen CH, Pedersen MM, et al. Implementation of neck/shoulder exercises for pain relief among industrial workers: a randomized controlled trial. *BMC Musculoskelet Disord* (2011) **12**:205. doi:10.1186/1471-2474-12-205
